# The evolving landscape of oral biology education: a comparative study of teaching strategies

**DOI:** 10.1186/s12909-025-07338-w

**Published:** 2025-05-26

**Authors:** Israa Ahmed Radwan, Sara El Moshy, Dina Rady, Nahed Sedky Korany, Fatma A. N. Abouel Maaty, Fatma I. Elfaiedi, Liza M. Monir, Mariam S. Ibrahim, Mai A. Ragab, Nada M. R. Osman, Nesma A. M. Shehata, Yasmin M. El-Ghazawy, Samah S. Mehanny, Marwa M. S. Abbass

**Affiliations:** 1 Oral Biology Department, Faculty of Dentistry, Cairo University, Cairo, Egypt; 2 Oral Biology Department, Faculty of Dentistry, Sirte University, Sirte, Libya; 3 Oral Biology Department, Faculty of Dentistry, Misr International University, Cairo, Egypt; 4 Oral Biology Department, Faculty of Dentistry, Fayoum University, Faiyum, Egypt; 5 Oral Biology Department, Faculty of Dentistry, Ahram Canadian University, 6 th of October City, Egypt; 6 Oral Biology Department, Faculty of Dentistry, Beni Suef University, Beni Suef, Egypt; 7 Department of Oral Biology, Faculty of Oral and Dental Medicine, Future University, Cairo, Egypt; 8 Oral Biology Department, Faculty of Dentistry, Cairo and Galala Universities, Cairo and Suez, Egypt

**Keywords:** Oral biology, Impact, Teaching methods, Dentistry teaching strategies

## Abstract

**Background:**

With the enormous changes in dental education to prepare well-trained dentists for future requirements, little is known about students’ feedback and their perceptions of their curriculum. With respect to academic progress, bridging the gap between basic biological sciences and clinical studies has recently become one of the prominent approaches. However, the perspectives of medical students are not frequently considered during the revision of the medical curriculum. This study aims to investigate the influence of different oral biology and embryology teaching strategies on dental students’ clinical awareness and practices.

**Methods:**

This cross-sectional study provided a structured and anonymous online questionnaire to at least 222 currently registered dental students and dental alumni from various Egyptian universities. The questions referred to oral biology teaching methods, clinical relevance, the use of virtual microscopes, and the associations of oral biology and embryology with other subjects in the dental curriculum. The answers were collected, and the data were statistically analyzed.

**Results:**

According to data, students strongly preferred modern teaching techniques over traditional ones, such as 3D holography and virtual microscopy. Most participants from both national and private universities expressed a strong preference for modern teaching techniques. The majority of participants in the study also concurred that oral biology topics are pertinent to clinical decision-making.

**Conclusion:**

By incorporating innovative technologies such as digital scanned slides, virtual microscopy, and holography, dental institutions can boost the educational process, especially the online process, and equip future dentists with essential skills for clinical excellence. Perpetual evaluation of teaching strategies is crucial for optimizing student engagement and ensuring the effectiveness of these advancements.

**Supplementary Information:**

The online version contains supplementary material available at 10.1186/s12909-025-07338-w.

## Background

Oral biology, a cornerstone discipline in dentistry, is the area of knowledge that deals with the structure, development, and function of oral tissues, providing the foundational knowledge necessary to understand the intricate relationship between oral health and systemic well-being [1]. The rapid emergence of oral biology as a discipline during the last two decades has occurred because of the compelling need for biological science teaching related to dentistry, research programs that lead to a better understanding of oral biological systems, and the discovery of new and improved methods for diagnosing, preventing, and controlling oral disease. By focusing on the traditional basic health sciences, oral biology has laid a foundation for the further development and expansion of the knowledge base upon which the clinical subjects of the dental curriculum and dental practice necessarily rest [2].

### Traditional teaching in oral biology

Dental education has traditionally relied on a blend of didactic lectures and practical demonstrations to deliver knowledge of oral biology. The didactic learning approach of oral histology materials involves knowledge transfer through classroom lectures and practical demonstrations of glass slides under the microscope [3]. While this approach has effectively imparted foundational knowledge, it often fails to inspire critical thinking and foster a deep understanding of the complex interplay between oral health and systemic well-being [4]. Effective teaching strategies in oral biology are crucial for equipping dental students with the necessary knowledge and skills to provide optimal patient care. Dental students are taught to rely on an impressive array of materials to restore function and cosmetics. However, recapitulating nature requires more. Dental students should be encouraged to think critically and creatively beyond the boundaries of procedure-based formulaic care and to envision innovative solutions that prioritize patient well-being and future dental practice [5].

### Emergence of digital teaching tools

As current medical and dental students are digital natives, they gravitate toward modern electronic learning tools that provide fast and efficient feedback [4]. The concept of virtual microscopy, which uses digitized slides for histology and pathology education, dates back to 2004 [6]. Researchers at that time suggested that this technology, with over a decade of use, could replace or supplement traditional glass slides in teaching [6]. In 2019, Tauber et al. confirmed students’ preference in dentistry and medicine for virtual slides over conventional glass slides [7]. Furthermore, in 2022, it was demonstrated that virtual microscopy has deep-rooted higher acceptance and has improved diagnostic ability [8]. Additionally, in 2022, Francis et al. mentioned that virtual microscopy technology had been implemented in several medical universities internationally, and the data regarding acceptance and efficacy as a teaching–learning method has been variable, although largely positive [9]. However, bridging digital usage requires a multifaceted approach incorporating infrastructure development, equitable access to technology, and robust support systems for students and educators [10].

In addition to the classical teaching method, Ghuloum explored an additional teaching tool in 2010. He stated that three dimensions (3D) hologram technology is potentially an effective teaching tool that could reinforce the learning process [11]. As a generic talk, the hologram is a three-dimensional record of the positive interference of laser light waves [11]. In educational contexts, holographic technology integrates elements from both virtual and physical realms. Unlike Virtual Reality, which fully immerses users in a virtual environment, holographic technology projects virtual elements into the real world, allowing users to maintain awareness of their surroundings. Holographic technology can potentially transform undergraduate scientific education and medical training. It enables medical students and practitioners to develop essential skills and knowledge, offering a promising future for educational innovation [12–14].

Never-ending seeking untraditional teaching tools, additional trials were conducted to deliver basic sciences in suitable, beneficial, and exciting ways [7]. In 2024, Ruado and Cortez stated that interactive slide representations significantly enhanced students’ achievement in biology, with an increase in students’ scores. Interactive slide representations also improve students’ cognitive, emotional, and behavioral engagement [15]. The new technologies, such as artificial intelligence (AI) simulation-based training and simulation-based training, offer personalized learning experiences in treatment and diagnosis, data-driven clinical decision support, and radiographic image analysis by automation, thus enhancing dental education [16, 17]. However, challenges such as infrastructure costs and scalability persist, particularly for digital tools [17].

### Knowledge gaps and objectives

Oral biology teaching practices vary globally from country to country as a function of resources available, curriculum design, and pedagogies. For instance, technology-driven and inquiry-based learning are prevalent in developed nations, such as the United States and most European nations [18]. On the other hand, developing countries, such as Egypt, face challenges in using innovative methods with limited inclusion in dental curricula. While virtual microscopy, holography, and interactive tools have been explored in past studies, little is known about their combined impact on conventional and modern methodologies within resource-limited settings. Comparisons of student attitudes and the clinical relevance of oral biology within such settings are also scarce, leading to an evidence gap in how to optimize dental education most effectively globally.

This research fills these gaps by examining the effects of varied teaching methods—various lectures to online tools such as virtual microscopy and holographyon the clinical knowledge and practice of dental students and alumni in Egypt. In contrast to previous research into individual technologies or developed-world contexts, this paper introduces a novel regional perspective that compares traditional and modern strategies in an emerging nation’s context. This study examines students’ attitudes, awareness of present-day technologies, and evaluation of the relevance of oral biology to clinical decisions. It also targets making context-relevant contributions to worldwide endeavors to improve dental education.

## Methods

### Ethical considerations

This study was conducted in accordance with the Declaration of Helsinki ethical standards. Accordingly, Ethical approval was obtained from the Ethics and Research Committee of the Faculty of Dentistry, Cairo University (approval number 32–5–42). After the study objectives were explained and confidentiality was assured, data were collected anonymously through self-administered online Google form questionnaires to maintain participant privacy, and the questionnaires did not contain any personal identification data to ensure confidentiality.

### Sample size calculation

The sample size was calculated via Epi-Info software, version 7.2.5.0 (Center for Disease Control and Prevention, Atlanta, GA, USA). According to previous results [19], 82.5% of undergraduate dental students in Saudi Arabia agreed that oral biology is relevant. Considering a confidence interval level of 95% and a margin of error of 5%, the total sample size was estimated to be 222 participants. By the time the link to the questionnaire was deactivated, a total of 287 responses were received, and therefore a total number of 287 respondents were included in the study.

### Study design and participants

This cross-sectional survey assessed dental students’ perceptions of the importance of oral biology and its clinical relevance. The participants were third- and fourth-year dental students, interns, and alumni enrolled in various Egyptian universities (governmental, private, and national). The inclusion criteria included being 19 years or older, understanding the study objectives, and being willing to provide informed consent. The exclusion criteria included nondental students, individuals not affiliated with Egyptian universities, those who still had running oral biology courses, dental staff members, individuals under 19 years of age, and those who declined to participate.

### Data collection

Only fully completed questionnaires were included in the data analysis. Dental students and alumni at various governmental, private, and national Egyptian universities were invited to participate in this study. The participants were asked to complete a structured, anonymous online questionnaire via Google Forms. The questionnaire was validated as its questions were previously utilized in previous studies conducted among medical students in Portugal [20] and Jordan [21] and dental students in India [20, 22, 23]. The questionnaire comprises four sections: demographic information; data on participants’ age, year of study, university affiliation, and perceived importance of the teaching approach and study resources. The second section consists of eight, 5-options, Likert-scale questions (1 = strongly disagree, 2 = disagree, 3 = neutral, 4 = agree, 5 = strongly agree) that assess participants’ views on the significance of different teaching approaches and study resources. The third section handles the impact of oral histology on clinical practice and comprises 16, 5-options, Likert-scale questions (1 = strongly disagree, 2 = disagree, 3 = neutral, 4 = agree, 5 = strongly agree) that evaluate the influence of oral histology knowledge on various clinical dental specialties. Suggestions for improving oral histology teaching section includes four questions soliciting participants’ opinions on enhancing teaching methods, addressing challenges, and increasing clinical relevance. The full questionnaire is available as a supplementary file.

To ensure sequential completion, the participants were required to answer all the questions before proceeding to the next section.

### Statistical analysis

The participants were categorized according to age as follows: AI aged 19–20 years, AII aged 21–22 years, and AIII aged more than 23 years. They were also classified according to graduating university into governmental university participants (Cairo University, Ain Shams University, Beni-Suef University and Fayoum University), and into private and national universities participants(Future University, Galala University, Misr International University, Misr University for Science and Technology, New Giza University, and Al Ahram Canadian University). The participants were divided according to their education level into graduates, including alumni and interns, and into undergraduates, including both 3rd^−^ and 4 th-year bachelor’s degree students.

Furthermore, participants’ responses to questions regarding the preference of recent teaching methods versus traditional methods (6 questions) and regarding consensus on the impact of knowledge of variable oral and para-oral tissue histology on variable clinical decisions (16 questions) were marked, and a total score was calculated for each participant. According to each participant’s individual score, the preferences of recent teaching methods versus traditional methods were categorized into high preference for recent teaching methods (score value > 75%), moderate preference for recent teaching methods (score value = 50%−75%), and high preference for traditional teaching methods (score value < 50%). With respect to the consensus on the impact of knowledge of various oral and para-oral tissues histology on variable clinical decisions, participants were grouped into high (score value > 75%), moderate (score value = 50%−75%), and low (score value < 50%) consensus groups.

The data were statistically presented as frequencies (numbers) and percentages. Since the data were nonparametric, the Kruskal‒Wallis test was used to compare different categories (graduating universities and educational level) of scores for teaching method preference and scores for consensus on the impact of knowledge of various oral and para-oral tissues histology on variable clinical decisions between participants. While the chi-square test was used to compare the participants’ categories (graduating universities and educational level) in terms of their answer to the following questions: Have you ever heard about or used a virtual microscope?, Have you ever heard about or used a 3D hologram?, Do you agree on the impact of oral biology on your clinical practice?. *P* values of less than 0.05 were considered statistically significant. All the statistical calculations were performed via the computer program IBM SPSS (Statistical Package for the Social Science; IBM Corp, Armonk, NY, USA), release 22 for Microsoft Windows.

## Results

### Demographics

**Fig. 1:**
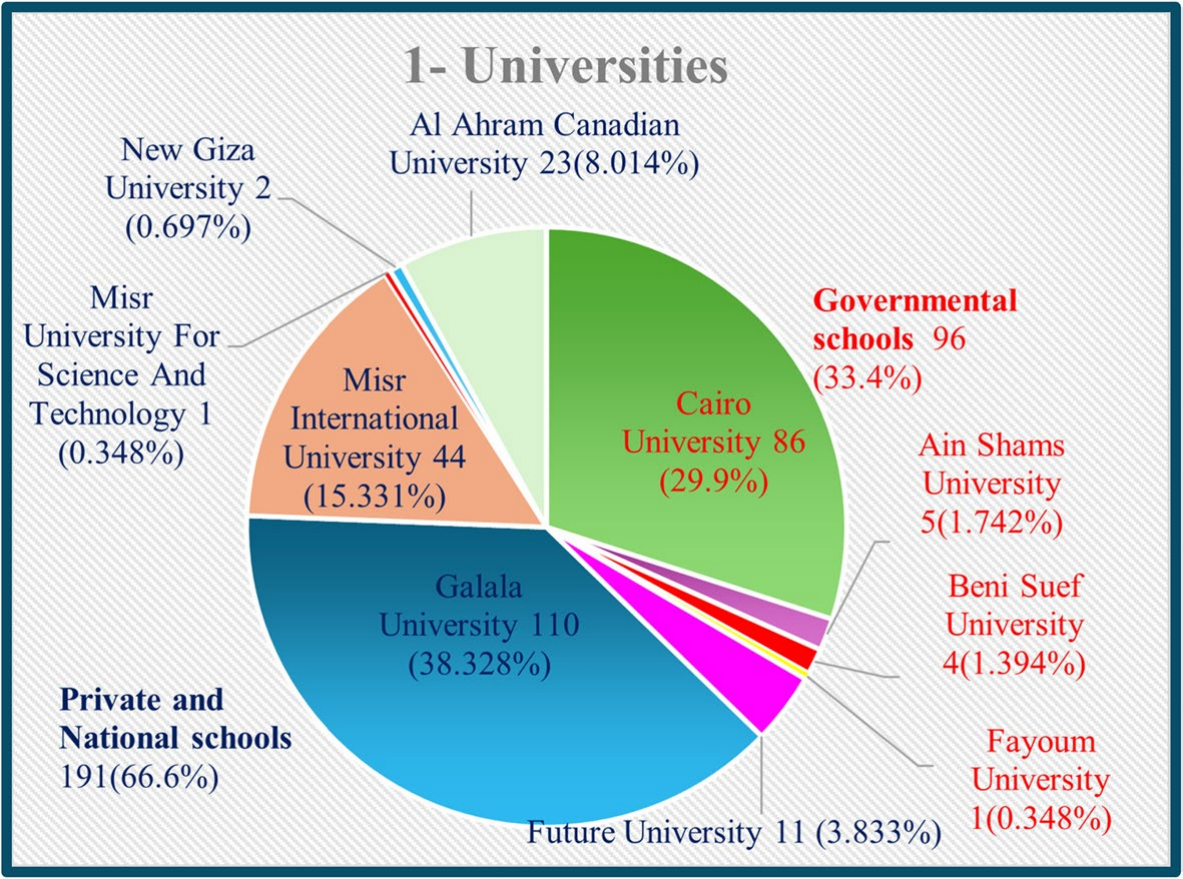
Pie chart illustrating the universities of participants

**Fig. 2:**
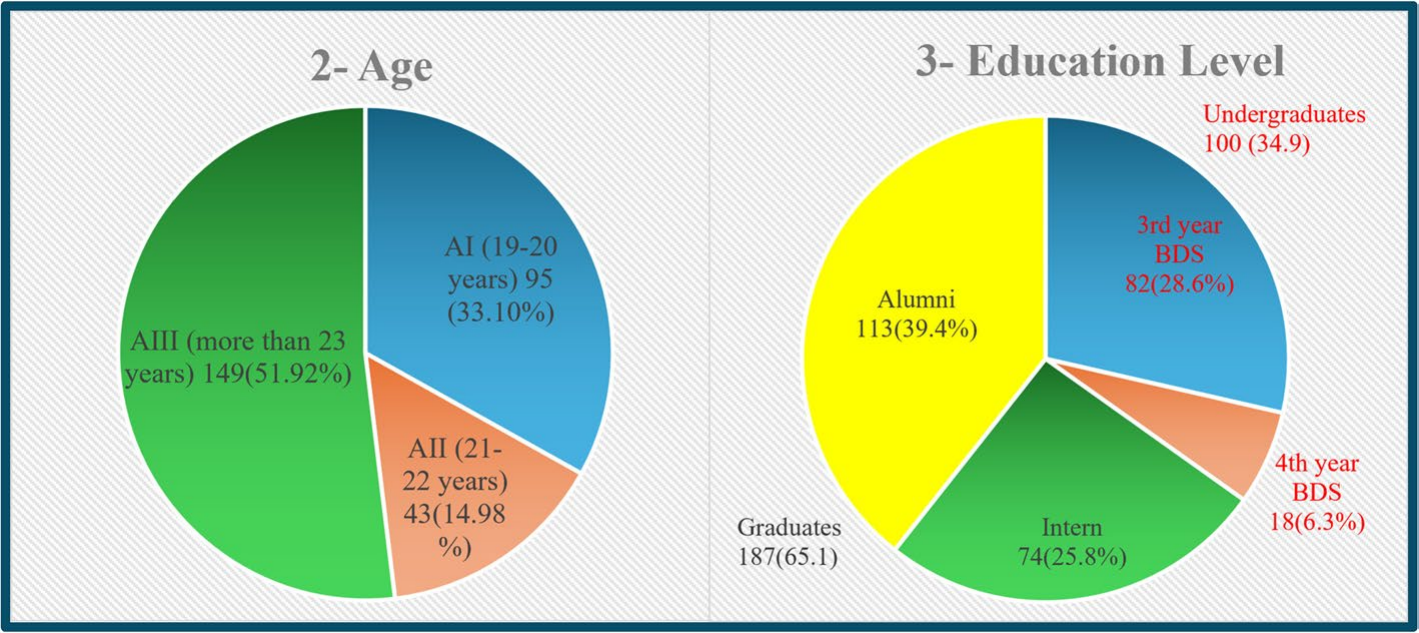
Pie charts illustrating **A** age of the participants and **B** educational level of the participants

A total of 287 participants were included in the current study. The participants belonged to various governmental, private, and national dental schools in different regions of Egypt. the current study included 96 (33.4%) participants from governmental universities and 191 (66.6%) from private and national universities across Egypt (Fig. 1). The sample included 95 (33.10%) participants aged 19–20 years, 43 (14.98%) aged 21–22 years, and 149 (51.92%) aged more than 23 years (Fig. 2A). The current study included 3rd-year students 82 (28.6%), 4 th-year students 18 (6.3%), interns 74 (28.8%) and alumni 113 (39.4%) (Fig. 2B).

### Teaching techniques

#### Educational teaching techniques

**Fig. 3:**
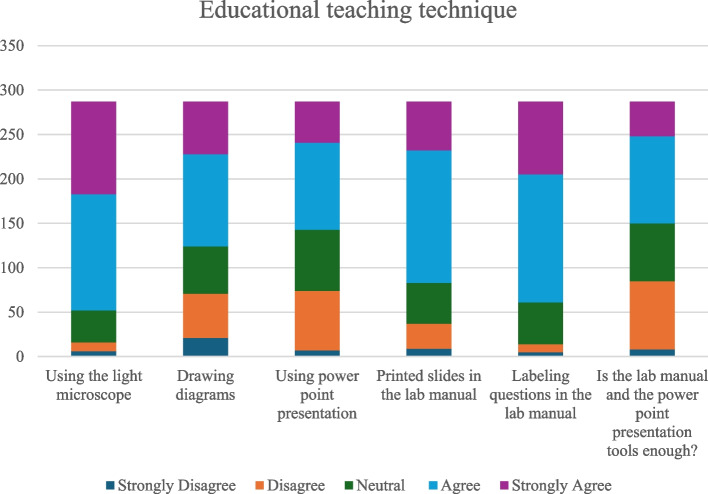
Bar chart summarizing participants’ responses to questions regarding their preference for teaching techniques

With respect to the educational technique questions, most of the participants agreed on the following: the importance of using the light microscope, the importance of drawing diagrams, the importance of using PowerPoint presentations, the importance of printed slides in the lab manual, and on the relevance of labeling questions in the lab manual. The majority of participants thought that the lab manual and the power point presentation tools were sufficient to understand and interpret oral histological slides (Fig. 3).

#### Score for participants’ preferences for recent teaching methods versus traditional methods applied in Egyptian schools of dentistry

The participants’ scores regarding their preference for recent teaching methods versus traditional methods applied in Egyptian schools of dentistry revealed that a greater percentage of participants 117 (40.77%), reported high preference for recent teaching methods (Table 1).

**Table 1:** Scores for participants’ preferences for recent teaching methods versus traditional methods applied in Egyptian schools of dentistry

**Score for teaching methods preference**		**High preference to recent teaching methods** 117 (40.77%)	**Moderate preference to recent teaching methods** 77 (26.83%)	**High preference to traditional teaching methods** 93 (32.40%)	***P* Value**
Graduating university and score for preference teaching methods	Governmental universities	28 (29.17%)	25 (26.04%)	43 (44.79%)	0.001^*^
	Private and National universities	89 (46.60%)	52 (27.23%)	50 (26.18%)	
Educational level and score for preference teaching methods	Bachelor’s degree students (3rd year BDS 4 th year BDS)	44 (44.00%)	30 (30.00%)	26 (26.00%)	0.172
	Graduates (alumni and interns)	73 (39.04%)	47 (25.13%)	67 (35.83%)	

*Statistically significant difference (*P* < 0.05)

The majority of participants belonging to private and national universities showed a high preference for recent teaching methods, while most of the participants belonging to governmental universities showed high preference for traditional teaching methods, with a significant difference between groups (*p* = 0.001) (Table 1).

The bulk of Bachelor’s degree students (3rd year BDS and 4 th year BDS) showed a high preference for recent teaching methods. Similarly, the majority of graduates (alumni and interns) showed a high preference for recent teaching methods in comparison to undergraduates (Table 1).

#### Knowledge about recent technologies in oral biology teaching techniques.

Most of the respondents mentioned that they had no prior knowledge of virtual microscopy 159 (55.40%) or 3D holograms 202 (70.38%). In contrast, 128 (44.60%) and 85 (29.62%) had prior knowledge about virtual microscopes and 3D holograms, respectively (Table 2).

**Table 2:** Participants’ opinions about the use of recent technologies in teaching oral biology

Recent technologies in teaching techniques				
Have you ever heard about or used virtual microscope?		Yes 128 (44.60%)	No 159 (55.40%)	*P* Value
Graduating university and knowledge about virtual microscope	Governmental universities	31(32.3%)	65(67.7%)	0.003^*^
	Private and National universities	97(50.8%)	94(49.2%)	
Educational level and knowledge about virtual microscope	Bachelor’s degree students (3rd year BDS 4 th year BDS)	58(58%)	42(42%)	0.001^*^
	Graduates (alumni and interns)	70(37.4%)	117(62.6%)	
Have you ever heard about or used 3D hologram?		Yes 85 (29.62%)	No 202 (70.38%)	*P* Value
Graduating university and knowledge about 3D hologram	Governmental universities	20(20.8%)	76(79.2%)	0.021^*^
	Private and National universities	65(34.04%)	126(65.96%)	
Educational level and knowledge about 3D hologram	Bachelor’s degree students (3rd year BDS 4 th year BDS)	42(42%)	58(58%)	0.001^*^
	Graduates (alumni and interns)	43(22.99%)	144(77.01%)	

*Statistically significant difference (*P* < 0.05)

**Fig. 4:**
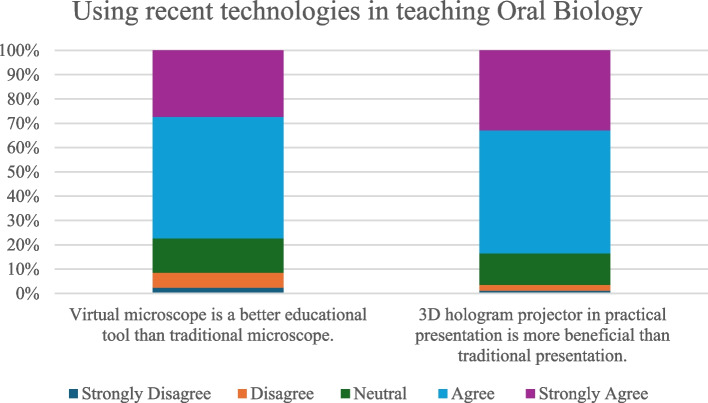
Bar chart summarizing participants’ opinions about the use of virtual microscopes and 3D holograms in teaching oral biology

Among the participants who responded positively, the majority of participants who had a previous knowledge about virtual microscope, and the majority of participants who had a previous knowledge about hologram projector in practical presentation agreed on their importance in in teaching oral biology (Fig. 4).

Ninety-seven (50.8%) of the respondents who belonged to private and national universities had prior knowledge about virtual microscopy, while 65 (67.7%) of respondents who belonged to governmental universities had no prior knowledge about the usage of virtual microscopes, with a statistically significant difference between groups (*p* = 0.003). 58 (58%) of undergraduates had prior knowledge of virtual microscopes, whereas 117(62.6%) of alumni had no prior knowledge of virtual microscopes, with a statistically significant difference between groups (*p* = 0.001) (Table 2).

The majority of respondents who belonged to private and national universities, and who belonged to governmental universities had no prior knowledge of 3D holograms, with a statistically significant difference between groups (*p* = 0.021). Additionally, the majority of the undergraduates and alumni had no prior knowledge of the 3D hologram, with a statistically significant difference between the groups (*p* = 0.001) (Table 2).

### Importance of oral biology in clinical decision-making and practice

#### The relevance of topics taught in oral biology and their clinical implications

**Fig. 5:**
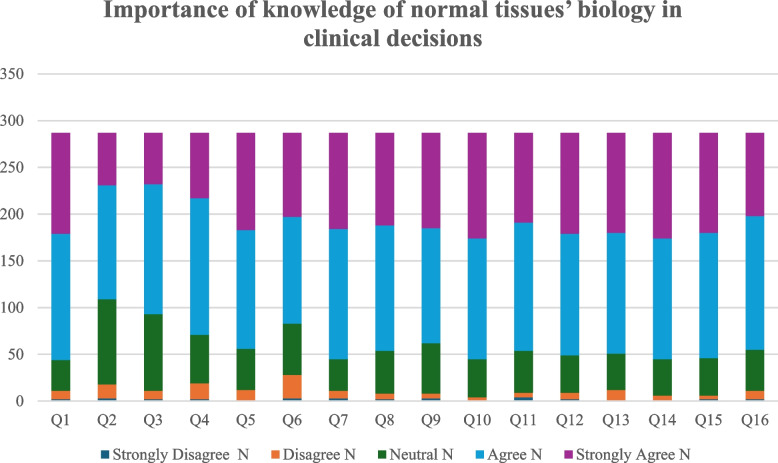
Bar chart summarizing participants’ opinions on the importance of knowledge of normal tissue biology in clinical decision-making

A greater number of participants agreed on the relevance of different subjects taught in oral biology and their relevance to clinical situations. The responses of participants to each of the given questions regarding the relevance of different oral biology related topics, is detailed in Fig. 5 and Table 3.

**Table 3:** Participants’ opinions on the importance of knowledge of normal tissue biology in clinical decision-making

Importance of knowledge of normal tissues’ biology in clinical decisions					
	Strongly Disagree N (%)	Disagree N (%)	Neutral N (%)	Agree N (%)	Strongly Agree N (%)
Q1 Regarding enamel and dentin structures and acid etchant	2 (0.70%)	9 (3.14%)	33 (11.50%)	135 (47.04%)	108 (37.63%)
Q2 Regarding dentin development and acid etchant	3 (1.05%)	15 (5.23%)	91 (31.71%)	122 (42.51%)	56 (19.51%)
Q3 Regarding dentin biology and the dentin-bonding mechanism	2 (0.70%)	9 (3.14%)	82 (28.57%)	139 (48.43%)	55 (19.16%)
Q4 Regarding dental hard tissues histology and cavity preparation	2 (0.70%)	17 (5.92%)	52 (18.12%)	146 (50.87%)	70 (24.39%)
Q5 Regarding enamel and dentin structures and preparation design	1 (0.35%)	11 (3.83%)	44 (15.33%)	127 (44.25%)	104 (36.24%)
Q6 Regarding enamel rod direction and caries spread	3 (1.05%)	25 (8.71%)	55 (19.16%)	114 (39.72%)	90 (31.36%)
Q7 Regarding oral soft tissue biology and smile design plan	3 (1.05%)	8 (2.79%)	34 (11.85%)	139 (48.43%)	103 (35.89%)
Q8 Regarding types of sensation in both the periodontal ligament and pulp in relation to differential diagnosis	2 (0.70%)	6 (2.09%)	46 (16.03%)	134 (46.69%)	99 (34.49%)
Q9 Regarding oral mucosa biology and periodontal surgeries	3 (1.05%)	5 (1.74%)	54 (18.82%)	123 (42.86%)	102 (35.54%)
Q10 Regarding alveolar bone biology and implant surgery was	0	4 (1.39%)	41 (14.29%)	129 (44.95%)	113 (39.37%)
Q11 Regarding osseointegration, soft and hard tissue biology and implant surgery	4 (1.39%)	5 (1.74%)	45 (15.68%)	137 (47.74%)	96 (33.45%)
Q12 Regarding orthodontic treatment requires and periodontium biology	2 (0.70%)	7 (2.44%)	40 (13.94%)	130 (45.30%)	108 (37.63%)
Q13 Regarding oral and para oral tissues biology and diagnosing pathological lesions	0	12 (4.18%)	39 (13.59%)	129 (44.95%)	107 (37.28%)
Q14 Regarding tooth development and diagnosing tooth anomalies	1 (0.35%)	5 (1.74%)	39 (13.59%)	129 (44.95%)	113 (39.37%)
Q15 Regarding maxillary sinus biology and implant placement and sinus lifting	2 (0.70%)	4 (1.39%)	40 (13.94%)	134 (46.69%)	107 (37.28%)
Q16 Regarding TMJ biology and diagnosis of TMJ disorders	2 (0.70%)	9 (3.14%)	44 (15.33%)	143 (49.83%)	89 (31.01%)

#### Score for participants’ consensus on the impact of knowledge of variable oral and para-oral tissues histology on variable clinical decisions

The participants’ scores regarding the consensus on the impact of knowledge of oral and para-oral tissues’ histology on various clinical decisions revealed that most of the respondents reported a high degree of consensus on the impact of learning oral biology on clinical decisions. The majority of participants from governmental, private, and national universities, Bachelor’s degree students and graduates shared a high consensus as to the importance of studying oral biology; however, the difference between different groups was not statistically significant (Table 4).

**Table 4:** Score for participants’ consensus on the impact of knowledge of various oral and para-oral tissues histology on variable clinical decisions

Consensus on the impact of knowledge of variable oral and para-oral tissues histology on variable clinical decisions		High 241 (83.97%)	Moderate 32 (11.15%)	Low 14 (4.88%)	*P* Value
Graduating university and score for consensus on the impact of knowledge of variable oral and para-oral tissues histology on variable clinical decisions	Governmental universities	78 (81.25%)	14 (14.58%)	4 (4.17%)	0.420
	Private and National Universities	163 (85.34%)	18 (9.42%)	10 (5.24%)	
Educational level and score for consensus on the impact of knowledge of variable oral and para-oral tissues histology on variable clinical decisions	Bachelor’s degree students (3rd year BDS and 4 th year BDS)	83 (83.00%)	10 (10.00%)	7 (7.00%)	0.671
	Graduates (alumni and interns)	158 (84.49%)	22 (11.76%)	7 (3.74%)	

A direct Yes or No question was directed to the participants “do you agree on the impact of oral biology on your clinical practice?” to which the greater part of participants replied with a Yes. Among the participants who replied with a Yes, 90 (32.61%) belonged to governmental universities, and 186 (67.39%) belonged to private and national universities. Additionally, among the participants who replied with a Yes, 97 (35.14%) were undergraduate students, whereas 179 (64.86%) were graduates. However, the difference between the groups was not statistically significant (Table 5).

**Table 5:** Student opinions on the impact of oral biology on clinical practice

Do you agree on the impact of oral biology on your clinical practice?		Yes 276 (96.17%)	No 11 (3.83%)	*P* Value
Graduating university and Student’s opinion on the impact of oral biology on your clinical practice	Governmental universities	90 (32.61%)	6 (54.55%)	0.143
	Private and National universities	186 (67.39%)	5 (45.45%)	
Educational level and Student’s opinion on the impact of oral biology on your clinical practice	Bachelor’s degree students (3rd year BDS and 4 th year BDS)	97 (35.14%)	3 (27.27%)	0.584
	Graduates (alumni and interns)	179 (64.86%)	8 (72.73%)	
If you partially or strongly disagree on the impact of oral biology on your clinical practice, what could be the reason?				N(%)
a. Lack of understanding or knowledge about oral biology				3(27.3%)
b. Different interpretation or perception on the role of oral biology in clinical practice				5(45.5%)
c. Varied professional experiences or trainings that have influenced your opinion				3(27.3%)
d. Limited exposure to updated data regarding the impact of oral biology on clinical practice				4(36.4%)
e. Differences in the emphasis or Importance placed on oral biology within individual clinical approaches or philosophies				4(36.4%)

Among the participants who replied with a No, 3 (27.3%) thought this was due to a lack of understanding or knowledge about oral biology. 5 (45.5%) thought this was because of different interpretations or perceptions of the role of oral biology in clinical practice. 3 (27.3%) thought it was due to varied professional experiences or training that influenced their opinions. 4 (36.4%) replied that this was because of limited exposure to updated data regarding the impact of oral biology on clinical practice. 4 (36.4%) pointed out that this was because of differences in the emphasis or importance placed on oral biology within individual clinical approaches or philosophies (Table 5).

## Discussion

This research highlights the vital importance of creative teaching methods in improving dental education, especially in oral biology. Traditional methods, while foundational, often fall short in engaging students and fostering a deep understanding of complex biological concepts. The integration of digital tools such as virtual microscopy and 3D holography has shown significant promise in bridging this gap.

A fundamental understanding of basic science is essential in dental practice to precisely diagnose patients and develop effective therapeutic strategies. Nonetheless, the importance of oral biology, with its branch of oral histology, in clinical training, practice, and dental education is not adequately acknowledged [24]. Moreover, it is crucial to assess the effectiveness of current teaching methods and evaluate whether traditional approaches need to be adapted or abandoned to enhance training and learning outcomes [25]. This is in conjunction with the significant increase in the number of dental schools in Arab countries, especially private institutions, due to the rising demand for dental education. However, due to salary disparities, dental education in Arab countries faces challenges such as inconsistent funding, increasing demand, and difficulty retaining faculty members [26].

The results of the present study indicate a strong preference among students for modern teaching methods over traditional methods. Similar findings were reported in Argentina, where 92% of the recruited students used the virtual laboratory as a principal tool for learning cell biology, histology, and embryology [27]. Moreover, compared with traditional optical microscopy, virtual microscopy has resulted in greater satisfaction among dental students during their oral histopathological studies [28]. On the other hand, this discovery contradicts the findings of previous studies [29–31], which indicated that most students favored lecture-based instruction. Unexpectedly, in Saudi Arabia, 86% of the students revealed their interest in using histological slides and compound microscopes for practical sessions rather than virtual histological images projected through PowerPoint presentations [24].

Students from private/national universities in the present work preferred modern methods, whereas governmental university students favored traditional approaches (*p* = 0.001). A lack of facilities and high student numbers in government universities contribute to this disparity. At the same time, better access to technological resources is likely available at private and national universities. Consistently, 50.8% of private/national university students were already familiar with virtual microscopes, compared to 67.7% unfamiliarity in government universities with a significant difference between groups (*p* = 0.003). This finding highlights the need for equitable access to educational technologies across all educational settings.

The implementation of virtual microscopy and 3D hologram technology within educational institutions is considered a turning point., yet, there are significant obstacles to adopting particularly in developing countries. The challenges include insufficient infrastructure and technology in many higher education institutions, limited internet connectivity, frequent power outages, a lack of technical knowledge, and a shortage of technical experts [32]. This is reflected in the fact that most of the participants in the current study lacked prior familiarity with virtual microscopes (55.4%) or 3D holograms (70.4%). On the other hand, 58% of undergraduates knew of virtual microscopes, versus 62.6% unfamiliarity among alumni (*p* = 0.001).This gap emphasizes the importance of introducing these technologies early in the dental curriculum to ensure that all students benefit from their advantages. The positive reception of these tools among students familiar with them (more than 50%) suggests that their broader implementation could enhance learning outcomes and better prepare students for clinical practice. The previous findings support the preference of another study for virtual microscopes because of their distinct features, which helped maintain uninterrupted quality education while adhering to social distancing and disease prevention guidelines during the COVID-19 pandemic lockdowns. Students reported positive effects such as self-paced learning, enhanced tissue recognition, better access to slides, improved comprehension, and greater academic achievement [33]. Moreover, a systematic review was conducted to examine the existing evidence on the effectiveness of virtual models versus traditional models for medical and dental school students’ learning, the results indicated a strong inclination toward the use of virtual microscopes [34]. Similarly, a meta-analysis concluded that students favor virtual microscopy over traditional light microscopy [35].

The gathered responses concerning the consensus on the impact of knowledge of variable oral and para-oral tissue histology on clinical decisions showed that 241 participants (83.97%) strongly agreed on the importance of understanding oral biology for making clinical decisions, with no statistically significant difference between groups. These results contrast with those of a previous study conducted in Saudi Arabia, where the majority of students found oral histology to be difficult and not very relevant to their future dental careers, and only a small number of students considered it very relevant [24]. Combining oral biology subjects with various clinical courses entailed a comprehensive grasp of the science, which encouraged the students and interns in the current study to identify the relevance between oral biology and the clinical disorders impacting the oral cavity.

The strong agreement on the importance of topics covered in oral biology and their relevance to clinical practice and decision-making in the current study aligns with a previous study conducted at King Saud University in Saudi Arabia, in which both undergraduate and postgraduate dental students considered oral biology relevant to dentistry, whereas postgraduate students reported greater relevance than undergraduates did [19]. Furthermore, in a study conducted by Shakeel et al. [36], the dental faculty and interns highlighted the importance of oral biology in dentistry. Oral surgery is the clinical dental department with the highest perceived relevance, as having a deep understanding of oral tissues and healing processes is crucial for future surgical procedures. Dental materials, the basic dental department, consider oral biology to be the most relevant dental subject, followed by community dentistry.

Since the oral biology course is image intensive, conventional methods of teaching oral histology via light microscopy and glass slides have several drawbacks, including the high cost of maintaining the microscope and the fading of color in the stained tissue sections on the slides over time [37]. Like all other educational systems, dental education is constantly advancing and evolving. For example, incorporating electronic/digital teaching methods [38] and software applications [39] has greatly increased the value of traditional histology teaching methods. The use of digital technology has empowered course managers, particularly in oral histology, to adjust teaching strategies efficiently, helping students conform to the continuously growing learning environment [40]. The findings of the present study align with those of previous studies in which a large portion of students reported never or rarely using textbooks, instead favoring electronic learning resources such as online videos and slides [24], indicating that innovative teaching methods significantly enhance students’ academic performance and engagement. Even for examination, the PowerPoint microscopy image was perceived as an easier and preferable tool for exams over traditional microscopy images among dental and medical students in Taiwan; however, the majority of students emphasized the fairness of the traditional method [41, 42]. Similarly, 3D holography offers an immersive learning experience that can enhance students’ spatial understanding of complex anatomical structures. The virtual slides offer the students high-quality images to be accessed and reviewed using image processing software for free, as long as they have access to broadband internet, without restricting time and space [34].

Zaletel et al. [43] determined that students in the Histology and Embryology course require increased clinical integration and a greater emphasis on incorporating clinical cases. Moreover, Gribbin and his team [44] suggested that introducing curriculum modifications with a more contemporary approach could significantly influence students’ academic success in histology. In China, reforming teaching methods by incorporating clinical practice with dental theory through problem-based learning on real clinical cases significantly improved dental students’ abilities to treat and communicate with patients [45]. Introducing serious histology games as a learning oral histology strategy for dental students was found to significantly improve students’ cognitive skills, and students who completed the game missions had higher posttest scores [46].

Although the findings of this study may not be generalizable to all the dental schools not involved in our research, they do provide potentially helpful insights. Future collaboration with more dental schools with a greater focus on investing in research and including technology in the learning process is mandatory. This technology helps dental students clarify these concepts in a better manner and enhances learning outcomes.

Among the limitations of this study, the questionnaire design that combined two distinct concepts, participants’ knowledge of the virtual microscope and hologram (cognitive level: “heard of”) and their actual use (behavioral level: “used”), into a single question. A step-by-step approach, separating these dimensions, would have strengthened logical consistency and data interpretation.

Additionally, the exclusion of dental staff may somewhat limit the generalizability of the findings to all stakeholders in oral biology education. However, this focus was intentional, as the study prioritized the perspectives of learners directly impacted by e-learning challenges. Shifting to e-learning is a critical issue in medical education, due to the high costs associated with hiring qualified instructors, developing digital resources, and maintaining infrastructure [47]. Future research should include dental staff to provide a more comprehensive evaluation of teaching strategies.

Furthermore, the study did not examine the potential of AI in enhancing creative teaching methods. AI-powered tools could personalize learning, provide automated feedback, and offer immersive simulations that potentially improving oral biology education. Investigating AI’s role in supplementing traditional methods is a valuable direction for future studies.

Finally, the assessment of creative teaching techniques relied primarily on qualitative feedback and student outcomes, which may introduce subjectivity. Using standardized evaluation tools or AI-driven analytics in future work could yield more objective insights.

## Conclusions

In summary, this research highlights the necessity for a change in dental education towards more creative and technology-focused instructional approaches. This could be applied through the complete substitution of glass slides into digitally scanned slides and by incorporating virtual microscopy and holography in the development of a comprehensive online learning platform for oral biology. Faculties should conduct continuous surveys and evaluations to improve online educational services to sustain positive reception by the majority of students. From this perspective, educational institutions can better equip future dentists with the oral biology knowledge and skills necessary to excel in their clinical practice. Future research should focus on exploring the long-term impacts of these teaching methods on students’ clinical performance and patient outcomes.

The rapid expansion of dental schools, especially within the private sector, highlights an increasing need for dental education alongside significant issues in funding stability, faculty retention and maintaining educational quality. To address these issues effectively, dental schools need to take decisive action by investing in attractive faculty salaries and professional development programs to draw in and keep skilled instructors. Simultaneously, using cost-effective technology-driven teaching approach can help maintain educational quality despite even with resource limitations. Enhancing accreditation systems and promoting public-private collaborations will be vital for achieving sustainable development while preserving educational standards. Through these coordinated efforts, academic leaders and policymakers have the potential to turn existing challenges into opportunities to enhance dental education regionally, thereby achieving the goal of training highly skilled dental professionals capable of meeting society’s healthcare needs.

## Supplementary Information

Supplementary Material 1.

## Data Availability

All data generated or analyzed during this study are included in this article and its supplementary information files.

## Abbreviations

3D: Three dimensions
BDS: Bachelor’s degree students
