# Supplementary material for: The evolving landscape of oral biology education: a comparative study of teaching strategies
Source: BMC Med Educ. 2025 May 26;25:779. doi: 10.1186/s12909-025-07338-w (PMC12107733; doi:10.1186/s12909-025-07338-w)
Supplement: Supplementary file 1 — Supplementary Material 1. [file 12909_2025_7338_MOESM1_ESM.docx]

Annex 1
-This study was approved by the Research Ethics Committee, Faculty of Dentistry, Cairo University. The questionnaire is intended to examine how studying oral biology as a pre-clinical course could serve clinical practice. The questionnaire is anonymous and intended for research purposes only. The information will be entered into the computer and analyzed along with all other responses. We would be very grateful if you could devote a few minutes of your time to filling out this questionnaire. Your responses are very important to us. Your participation is voluntary, and you are free to stop filling the questionnaire at any time.

**Section 1: Demographic information**

**Age (years)***

a. 19-20

b. 21-22

c. ≥23 years

**Year of study***

a. 3rd year BDS

b. 4th year BDS

c. Intern

d. Alumni

**University***

Your answer:

**Section 2: Questions focusing on perceiving importance for teaching approach and study resources.**

**1-Do you think that using the light microscope makes studying oral biology a good experience?***

a. Strongly disagree

b. Disagree

c. Neutral

d. Agree

e. Strongly agree

**2-Do you think that power point presentation displaying the histological slides is a better alternative to the microscope?***

a. Strongly disagree

b. Disagree

c. Neutral

d. Agree

e. Strongly agree

**3- Do you think that drawing diagrams for the histological slides is beneficial?***

a. Strongly disagree

b. Disagree

c. Neutral

d. Agree

e. Strongly agree

**4- Do you think that the printed slides in the lab manual are considered a beneficial tool in studying oral biology?** *

a. Strongly disagree

b. Disagree

c. Neutral

d. Agree

e. Strongly agree

**5- Do you think that labeling questions in the lab manual are beneficial?** *

a. Strongly disagree

b. Disagree

c. Neutral

d. Agree

e. Strongly agree

**6- Do you think that lab manual and the power point presentation tools are enough to understand and interpret oral histological slides?** *

a. Strongly disagree

b. Disagree

c. Neutral

d. Agree

e. Strongly agree

**7- Have you ever heard about or used virtual microscope as an explanatory tool in learning oral biology?** *

Yes

No

**8- Have you ever heard about or used 3D hologram while studying oral biology?** *

Yes

No

**Section 3: Questions focusing on perceiving the importance of studying oral biology in clinical practice.**

**9- Does the oral biological basis, concerning the differences in enamel and dentin structures, affect your decision regarding the type of acid etchant and the duration of its application on both tissues?** *

a. Strongly disagree

b. Disagree

c. Neutral

d. Agree

e. Strongly agree

**10- Does the oral biological basis, concerning the role of active-matrix metalloproteinases during dentin development and their inactive form in fully matured dentin, influence your decision to use acid etchant with anti-enzymatic activities to prolong the durability of resin-based restorations?** *

a. Strongly disagree

b. Disagree

c. Neutral

d. Agree

e. Strongly agree

**11- Was the oral biological basis, regarding the decrease in dentinal tubules' diameter occlusally, which induces wider inter-tubular dentin areas, the main reason for adopting the concept of a hybrid layer as a dentin-bonding mechanism?** *

a. Strongly disagree

b. Disagree

c. Neutral

d. Agree

e. Strongly agree

**12- As per your understanding of the oral biology, does the histology of the dentino-enamel junction influences cavity outline's extension cervically?** *

a. Strongly disagree

b. Disagree

c. Neutral

d. Agree

e. Strongly agree

**13- Does your background knowledge of the biology of different dental tissues affect your clinical practice in terms of the adhesive restorative concept? Specifically, influencing the preparation design, restorative material selection, and material placement technique?** *

a. Strongly disagree

b. Disagree

c. Neutral

d. Agree

e. Strongly agree

**14- As per your understanding of the oral biology, does the orientation of enamel rods influence your expectations for caries spread and affects your treatment plan, whether it be a traditional cavity, inlay, or onlay?** *

a. Strongly disagree

b. Disagree

c. Neutral

d. Agree

e. Strongly agree

**15- As per your understanding of the biology of the oral mucosa, does the macro-anatomy of the gingiva influence your smile design plan?** *

a. Strongly disagree

b. Disagree

c. Neutral

d. Agree

e. Strongly agree

**16- As per your understanding of the oral biology, does knowing different nerve receptors and various types of sensation in both PDL and pulp aid in differential diagnosis between their insult?** *

a. Strongly disagree

b. Disagree

c. Neutral

d. Agree

e. Strongly agree

**17- Does studying of the biological structure of oral mucosa help you during periodontal surgeries?** *

a. Strongly disagree

b. Disagree

c. Neutral

d. Agree

e. Strongly agree

**18- Does studying of the biological structure** **of the alveolar bone serve your clinical practice while performing implant surgery?** *

a. Strongly disagree

b. Disagree

c. Neutral

d. Agree

e. Strongly agree

**19- Does understanding of osseointegration and gingival cells behavior during tissue healing affect your treatment plan regarding the choice of implant material and the technique of insertion to achieve the best results?** *

a. Strongly disagree

b. Disagree

c. Neutral

d. Agree

e. Strongly agree

**20- Do you agree that orthodontic treatment regarding applying different types of forces requires well knowledge of the biological structure of periodontium?** *

a. Strongly disagree

b. Disagree

c. Neutral

d. Agree

e. Strongly agree

**21- Does your knowledge of the biological structure of oral and para oral tissues help you in diagnosing various pathological lesions?** *

a. Strongly disagree

b. Disagree

c. Neutral

d. Agree

e. Strongly agree

**22- Does studying tooth development and understanding how defects can occur during tooth development can help you in diagnosing tooth anomalies clinically?** *

a. Strongly disagree

b. Disagree

c. Neutral

d. Agree

e. Strongly Agree

**23- Does studying maxillary sinus development and microanatomy influence your clinical skills in performing safe extractions for upper teeth, implant surgeries and selecting sinus lifting approach?** *

a. Strongly disagree

b. Disagree

c. Neutral

d. Agree

e. Strongly agree

**24- Does your knowledge about the histological structure of TMJ affect your**diagnostic talents**in TMJ disorder cases?** *

a. Strongly disagree

b. Disagree

c. Neutral

d. Agree

e. Strongly agree

**Do you agree on the** **impact of oral biology on your clinical practice?** *

Yes

No

**Section 4:  Questions focusing on the students’ point of views, opinions and suggestions.**

**25- If you partially or strongly disagree on the impact of oral biology on your clinical practice, what could be the reason? 
You can choose more than one answer***

a. Lack of understanding or knowledge about oral biology.

b. Different interpretation or perception of the role of oral biology in clinical practice.

c. Varied professional experiences or training that have influenced your opinion.

d. Limited exposure to updated data regarding the impact of oral biology on clinical practice.

e. Differences in the emphasis or importance placed on oral biology within individual clinical approaches or philosophies.

**26- In your opinion, what could be done to modify the** **oral biology curriculum to make it more clinically related?**

Your answer:

**27- In your opinion,** **what are the current challenges in integrating the oral biology into clinical practice?**

Your answer:

**28- From your own point of view, what do you think can be done to make the oral biology more interesting** **practically and theoretically?**

Your answer:
